# The Dynamic Interaction of Second Language Motivation and Emotional Experience: The Case of Chinese Learners of English

**DOI:** 10.3389/fpsyg.2022.905429

**Published:** 2022-06-06

**Authors:** Lixian Tian, Steven G. McCafferty

**Affiliations:** ^1^College of Teacher Education, Hebei Normal University, Shijiazhuang, China; ^2^Department of Educational Psychology and Higher Education, College of Education, University of Nevada, Las Vegas, Las Vegas, NV, United States

**Keywords:** motivation, goal-directed activity, *perezhivanie*, Chinese learners of English, emotion

## Abstract

This study investigated the dynamic interaction of second language motivation and emotional experience for Chinese learners of English in four different contexts: (1) those in China with little or no intention of learning the language for communicative purposes; (2) those in China intending to study abroad; (3) those studying abroad in North America; and (4) those who had returned to China after studying abroad. Data included interviews with representatives from the different contexts. Analysis focused on Vygotsky’s concept of *perezhivanie*, or how Chinese learners of English experienced their learning environments cognitively and affectively as leading to goals and goal-directed activity across their English learning experiences. Differences among learners both across groups and within the same group were found for how motivation shapes experience and how experience shapes motivation in the process of second and foreign language learning.

## Introduction

A major approach to second language L2 motivational research emphasizes studying specific learner behaviors in situated, dynamic contexts (e.g., De Bot et al., 2007; De Bot, 2008; Dörnyei, 2009; Dörnyei and Ryan, 2015). From this perspective, motivation is viewed in terms of complex systems and how different motivational constructs influence each other during development (Waninge et al., 2014; Dörnyei et al., 2015). Research on L2 motivation also has been undertaken from a sociocultural theory (SCT) perspective. Most SCT informed studies of L2 learning have focused on mediation in problem solving as an aspect of gaining self-regulation in classroom-focused research. However, there also have been efforts to view changes in motivation over time as related to goals and goal-directed activity as a result of changes in orientation (e.g., Lantolf and Genung, 2002), concentrating on the interaction of the person and their environment, a perspective we adopt in the current study. Emotion, although a relative newcomer to the study of second language acquisition (Prior, 2019) has been recognized as important in the study of language teaching and learning (Aragão, 2011; Dewaele, 2015; Poehner and Swain, 2016; Poole and Huang, 2018; Benesch, 2019; Bigelow, 2019; Dewaele, 2019; Kong, 2019; Lantolf and Swain, 2019; Bielak and Mystkowska-Wiertelak, 2020; Wang et al., 2021). We emphasize the role of emotion in development as related to Vygotsky’s concept of *perezhivanie*, or how a person experiences an event or environment through “refractive” (interpretive) processes that are both cognitive and affective (Vygotsky, 1994, p. 342).

This study focuses on both English as a foreign language (EFL) and English as a second language (ESL) adult learners, and although EFL students have been studied before, the subgroup of learners preparing to go abroad has not. Additionally, the study includes students who were in the process of pursuing a degree at an institution abroad where English was the dominant language, and the fourth contexts included is novel to the present study – former study-abroad students who had returned to China. Data are analyzed from a SCT perspective in order to gain insight into L2 motivation as related to how participants experienced their different language learning environments in relation to their goals and goal-directed activity.

## Literature Review

### Sociocultural Motivation and Activity in Second Language Development

Vygotsky viewed activity from the perspective of mediation as both practical and psychological (dialectical materialism) and as grounded in particular cultural-historical contexts, highlighting the dynamic, and dialectical interdependence of individuals and society (Vygotsky, 1978, p. 97). Psychological mediation goes together with agency, motivation, and phenomenal experience, something Vygotsky was concentrating on in relation to his concept of *perezhivanie* as a unit in the study of consciousness (Yasnitsky and van der Veer, 2016). *Perezhivanie* centers on personhood as a psychological whole, incorporating emotions, personality, motivation, fantasy (imagination), and cognition as dynamically interconnected (Blunden, 2016; Ferholt and Nilsson, 2016; Lantolf and Swain, 2019).

*Perezhivanie* represents a dynamic, person plus environment unity/unit as associated with socioculturally constructed leading activities (Kozulin, 2016) in the overall process of development. According to a SCT perspective researchers need to consider individual goals as critical to cognitive, emotional, cultural, and physical activity. Actions and goals are dominant features of human consciousness when interacting with the eco-social environment from an SCT perspective (Roth and Lee, 2007). Studying/learning a new language can be primarily a cognitive task involving study of the L2 as a grammatical system to be learned as curricular subject matter, which is the goal for many EFL learners in classroom environments. However, if goals include using the language for communicative purposes outside of the classroom, then the pragmatics of social interaction necessarily become a meaningful aspect of second language learning, especially when living and studying/working where the L2 is dominant. This environment makes the L2 more than just an artifact to be learned, which necessarily entails different goals and goal-directed activity in relation to agency. SCT agency [see Arievitch (2017), p. 33], especially for older children and adults, is critical in linking motivation to action/activity across environments and events. Learners can choose to fully embrace the new language and experiences they encounter, select which languacultural practices they wish to engage in or retreat back to the familiar contexts of first language and culture.

### Empirical Studies of Emotional Experience in Second Language Development

Previous SCT studies have focused on L2 motivation and activity, attending to changes in orientation as leading to emergent goals and goal-directed activity as connected to *perezhivanie*. For example, Lantolf and Swain (2019), reinterpreting the findings for a previous study, Lantolf and Genung (2002), focused on how the focal participant in the original study had shifted her motives and goals when studying Chinese as a foreign language as a graduate student in relation to *perezhivanie*. She had not found the instructional style or the curriculum conducive to learning the language for communicative purposes (her original goal), and after a period of frustration, had decided only to pursue a superior grade in the course (necessary to keep her scholarship). This change in orientation was connected to *perezhivanie* by Lantolf and Swain (2019) as leading to corresponding changes in goal-directed activity (a focus on class assignments and exams).

In another longitudinal study, Coughlan and Duff (1994), found that asking the same participant to respond to the same picture elicitation task 2 years and 2 months later produced quite different results. On the second iteration of the task the participant appeared less fluent in the L2 and less confident as well, despite having continued to study the L2 and having the same interlocutor and instructions as at time one. The researchers attributed this change to the difficulties the participant had in re-orienting to the task and interlocutor, that is, trying to balance interactive roles as well as finding a way in which to frame her description of the picture, which affected both intra- and interpersonal levels of meaning-making, the same task having become a different activity, emergent goals having changed as a consequence of *perezhivanie*.

In another study, Imai (2010) regarded emotion as socially constructed and mediated acts for EFL students engaged in collaborative tasks in which students formed emotional intersubjectivity in the process of co-constructing goal-directed activity. Additionally, Mahn and John-Steiner (2002) examined L2 writing as mediated collaborative activity through the lens of *perezhivanie* and Zone of Proximal Development, finding that L2 leaners provided emotional scaffolding, which impacted student cognitive performance as well. Poehner and Swain (2016) also studied mediated development and L2 writing, noting the cognitive-emotive unity of *perezhivanie*. From another perspective on *perezhivanie*, Cross (2012) examined how Japanese L2 students mediated learning during geography course work in content and language integrated learning (CLIL) contexts. This study viewed *perezhivanie* as the affective core of creativity, both teacher and students actively creating something new in the process of meaning-making through examining the language use and subject content found in the study of geography. Students regarded the CLIC approach as being “active” and “new,” and allowing them to take ownership of the language and learning. In an additional study, Garratt (2012) reported on how *perezhivanie* changed students’ affective experiences in relation to peer-to-peer text chat, that the experience increased their confidence in non-academic English writing, reducing their anxiety, text chats becoming welcome as “friendly,” “enjoyable,” and “motivating.” In addition, Pavelescu (2019), examined the intertwined link between motivation and emotion with two contrasting cases of EFL students’ learning experiences. One student felt positive emotions toward English learning, which enhanced motivation, while the other did not report a dominate emotion toward English, which hindered motivation. Moreover, differences in the two students’ emotional experiences led to different L2 future goals.

Gesture is another form of mediation that relates to *perezhivanie* and L2 learning. For example, Peltier and McCafferty (2010) found L2 learners of Italian in intermediate and advanced university courses deployed many gestures specific to the Italian languaculture. When interviewed, students mentioned their awareness of how gestures have a meaning-making role in the language and culture through watching and interacting with their instructors, who actively gestured in an Italian manner. Students wanted to incorporate gesture and embody the language as connected to possible future selves as Italian speakers. For example, one student stated that he not only wanted to learn Italian but wanted to “be” Italian in the coming together of motivation, goals, and *perezhivanie*. Additionally, Tian and McCafferty (2021) conducted a longitudinal study to examine Chinese international students’ multicultural identity in conjunction with co-speech gesture awareness and use while studying abroad. Two participants’ L2 gesture appropriation was found to change over time as an aspect of immersion in the American culture in conjunction with emotional conflicts related to identity, conscious agency affecting their awareness and use of co-speech gesture as tied to *perezhivanie*.

### Motivation of Chinese Learners of English

Previous studies have examined the motivation of Chinese learners of English, finding the influence of family, future employment, job promotion, academic achievement, and “face” to be culturally important (e.g. You et al., 2016). For instance, Magid (2009) found that both middle school and university student motivation was affected by family influence. Taguchi et al. (2009) also noted the influence of family on motivation, both with regard to doing well at school and in relation to job promotion. Additionally, You and Dörnyei (2014) highlighted the influence of the Chinese achievement “mindset” and the cultural concept of “face” as affecting motivation. In a longitudinal study concerning L2 motivation, Li and Ruan (2015) conducted mixed method research on changes in beliefs about English learning among Chinese learners of English over a period of 1 year, finding that content matter, extracurricular activities, assessments, and teachers all contributed to changes in motivation. Furthermore, Chinese learners of English orientation toward future selves has been found to focus on student desire to do well on exams and reach career and social goals, which in turn, affected L2 learning efforts and outcomes (Warden and Lin, 2000; Wu, 2001; Gao et al., 2004; Chen et al., 2005; Liu, 2007; Chen and Wu, 2011; Li and Ruan, 2015; Heying and Kennedy, 2016).

With regard to the study of motivation and Chinese learners in different L2 contexts, Chang (2018) explored non-English major’s motivational dynamics in EFL contexts through the use of dynamic system theory, finding both environmental (society, university, and English classroom instruction) and psychological considerations (interests and anxiety) affected motivation to learn the language. Additionally, Yuan and Ji (2017) found that English proficiency, teaching materials, teachers’ use of English, and access to smart phones affected motivation for four Chinese college students’ EFL in-class motivation using a microgenetic approach. Studies have also compared motivation as found for Chinese learners in EFL and ESL contexts. For example, Li (2011, 2014) found that ESL learners in New Zealand had higher motivational dispositions related to ideal L2 self, more positive attitudes toward learning English, higher levels of intended effort, and were less exam-oriented than EFL students in China. Moreover, ESL participants who had been in New Zealand for an extended period of time were found to have a more developed sense of ideal L2 self than recent arrivals, findings related to both contact with L2 speakers and amount of time spent in country. In another study, Yu et al. (2018) investigated motivation in relation to possible selves, focusing on 20 Chinese students in both EFL and ESL contexts, finding that ESL learners viewed oral communicative competence as more important than learners in EFL contexts in the dynamics of self-identity development and environmental exposure.

## Methodology

This qualitative research used a collective case study design (Creswell, 2007, p. 73) in examining the dynamic interaction of motivation and emotional experience (*perezhivanie*) for Chinese learners of English in four different learning environments, focusing on how the interplay of linguistic, social, and cultural dimensions of experience affected motivation to learn English. Selected participants from each group were interviewed concerning what they thought motivated them to learn the L2 and how their goal-directed activity was affected by their *perezhivanie* in the process.

### Research Questions

1. What are the differences in motivation for Chinese learners of English in the four different contexts of exposure?
2. How do goals and goal-directed activity as affected by *perezhivanie*, and as reported by selected participants in each group, affect L2 motivation?

### Participants

All participants had grown up as members of the Chinese culture in China. Mandarin was their first language and English was their foreign or second language. Participants (all names are pseudonyms) were selected from universities in China at different levels of academic standing and at different levels of English proficiency within each group in order to provide a representative sample. The first EFL group (group 1) consisted of college students in China studying English with no intention of studying abroad. The second EFL group (group 2) was studying English at both public universities and private tutoring schools to prepare for a study-abroad or work-abroad experience. The first ESL group (group 3), consisted of international university students studying in North America at the time of the study for degree purposes. The second ESL group (group 4), after being exposed as ESL learners to living and studying abroad, had returned to China and either did or did not continue to use English for academic studies or employment purposes. Groups 1 and 3 consisted of four participants each and groups 2 and 4 of three.

### Data Collection

In the process of data collection the principal researcher video-recorded semi-structured individual and small group (up to 3 participants) interviews. Interview questions focused on motivation and goal-directed activity as related to *perezhivanie*, changes in learning goals and the reason(s) why, feelings while speaking English in different situations, attachment to their English-speaking community, and perceived improvement over the course of their L2 learning experiences. Participants were also asked to describe their daily life experiences and were free to use either Mandarin or English during responses. Interviews lasted from 40 to 60 min each.

### Data Analysis

MAXQDA software (version 12) was used to analyze the interview data. Reported SCT activity related to emergent goals and goal-directed activity as linked to motivation and as an aspect of *perezhivanie* were analyzed. Following Lichtman’s (2012) model of data analysis: first we coded each participant’s response individually; then re-examined the list of categories for fit; and finally, undertook cross-group and within-group comparisons. Qualitative findings yielded between and within group similarities and differences for *perezhivanie* as affecting motivation and motivation as affecting *perezhivanie* with themes of goal-directed activity and affective reactions to activity.

## Findings

### Group 1

#### Profile

All four participants were undergraduate males, aging from 19 to 20. Kai and Ke were seniors, studying in a capital city in a northern province which afforded them only limited opportunities to communicate in English. The other interviewees, Ming, a senior and Yong, a sophomore, were studying in Beijing at the same university. At their university, international scholars were invited to give lectures in English almost daily and taught short-term courses, but the two students thought that “*such opportunities are more for graduate students*” (Ming). All four participants had decided not to study abroad in their near future.

#### Goal-Directed Activity

Interviewees had begun preparing for the English exam needed to be admitted to graduate school. Ming planned to enroll in tutoring classes in Beijing and to read English texts. Ke and Kai indicated that they had little access to quality tutors in their location but instead watched video-recorded tutorials online. Kai had also joined the English club at his school, but reported that: *“Members only focused on increasing vocabulary and rote learning.”*

In addition to the current necessity of learning English for exams, Kai and Ke expressed an obligation to locate “first-hand information” through reading articles and other materials written in English in order to stay abreast of their areas of study: “*It is better to read “firsthand” English literature in the future. If you read “second hand” definitely, it will be worse than what you understand.”* (Kai).

When asked about their future goals for learning English Ke and Kai said they wanted to be able to “*show off in front of those who were better at English [than me before]”* (Ke) and to be able to communicate in English when traveling, (a goal also stated by the Beijing students). They also wanted to be able to “translate books.” However, neither of the interviewees mentioned any emergent goals or goal-directed activity that would support these ambitions, instead the comments seemed to be off-the-cuff, and somewhat ludic. Moreover, they felt that the effort they had put into learning English in high school had been “the peak” of their English learning experience, saying that they “*did not work harder on English than in high school*” or even “*went backward from high school*” (Kai) while learning in college.

The two participants studying in Beijing, Ming and Yong, said that they did not speak English often, except for occasional communication in daily life, despite many foreigners working and studying at their university and in the larger area around them. Ming had once attended a lecture given in English but left in the middle feeling frustrated with his inability to “*comprehend the language*.” Also, Ming mentioned that when he encountered English native speakers, they could understand and speak Chinese and preferred to practice their Chinese instead of helping him to practice English.

#### Affective Reactions

Participants in this group were not confident about their English and thought it would take a long time to become fluent. Kai realized the difference between himself and “good” English learners:

*Those good English learners could speak English fluently and their pronunciation and intonation are pretty good. It seems that they are happy while speaking English. However, when I am speaking English, it is just “I am XX”, very awkward.*

Kai and Ke felt too ashamed to speak English in the interview. They only haltingly said a few words in English while describing an experience they had watching movies. They suggested the importance of continued efforts to learn the language, but did not mention any specific plans to do so.

Unlike the other participants in this group, Yong said that he was eager to study English and that he had attended English courses at other universities. Although he genuinely expressed interest in studying the language, wanting to communicate “*accurately*” and “*elegantly*,” his desire to “*do well in school*” took president.

### Group 2

#### Profile

Two of the three participants in group 2, Bei and Deng, aged 21, were awaiting offers to study abroad for a master’s degree. Deng had started to prepare for the TOEFL in high school and had attended tutoring courses during winter and summer breaks. Bei had enrolled in many English tutoring courses to prepare to study abroad. Once abroad, Bei planned to learn English for half a year before starting to take major-specific courses. The other member, Shi, in his early 30s, was employed as a post-doc at a research institute and planned to work for international companies or pursue another degree abroad. All three participants were male.

#### Goal-Directed Activity

The goal of living abroad had emerged from prior experience for this group, all of whom had traveled or attended conferences, circumstances in which face-to-face interaction in the L2 was central to the experience. Deng had had an internship with an international company in China and had volunteered during a summer break to help native-speaking English teachers in Beijing. He mentioned that he had learned to adapt to speaking English with native speakers after several days of communication during his internship and volunteer experiences. Bei had worked as a translator for two British tennis coaches during an international competition held in China and had traveled outside of China with his parents. Shi had attended international conferences where English was the medium of communication.

Goal-directed activity for these participants at the time of the interview centered on developing the L2 for educational/work related goals. All of them were conscious of the need to improve proficiency, which in addition to taking courses, included reading books and watching movies and TV shows in English (goal-directed activity) as aimed at achieving fluency in the language. Also, all three participants were receiving tutoring in Beijing at private schools, although for different purposes: Bei and Deng wanted to pursue a master’s degree abroad, concentrating on English exams for that purpose. Shi, on the other hand, wanted to concentrate on overall English proficiency in more general terms.

When reflecting on recent learning outcomes, Deng and Bei believed that their English had improved in helping them prepare for the TOEFL and other such exams, but as also stated by Shi, all participants realized they still had a long way to go, recognizing the discrepancy between their current levels of proficiency and what might be needed to effectively study/work abroad. In particular, all specifically mentioned a need to concentrate on English “lexical equivalents” for terminology as related to their interests, work, and/or academic fields.

In addition to the efforts to learn English mentioned above, Bei had recently decided to take a package of courses at the same studying-abroad agency (tutoring school) he had been attending up to the point of the interview and as based on his mother’s advice. He also had applied for “bridge courses” directed at improving English at the university he wished to attend for his study abroad experience, which entailed taking L2 English classes for one semester before starting graduate study. He felt that being immersed in the environment before starting academic courses was a necessary step.

Different from the other two participants, Shi, as established above, wanted to work for international companies, which required higher levels of English proficiency than those for study-abroad purposes. He was more concerned about reaching a level of proficiency that would not limit his possibilities for career advancement. Although he had published articles in English and had attended international conferences, he believed near-native fluency was necessary in order to be truly successful. To this end, he had spent around 6,000 US dollars on a 3-month English course that met three times a week, but summed up the experience, saying, “*Learning English can provide more chances to communicate and a broader view*… *but I did not reach that level.*”

#### Affective Reactions

Participants in this group expressed positive dispositions toward both Western education and culture and had applied to American universities. Two had chosen English names after reading novels and watching movies in English, Deng choosing the name of one of his favorite characters in the Harry Potter series, and Bei choosing a name from an American movie he had watched the night before attending an L2 class at his university.

Bei also mentioned that he had become more confident after traveling abroad several times. At first, he was afraid of even trying to speak English, but on later trips realized that even if his English was not very well developed, he could understand what others said and how to reply to them:

*“I was forced to be an interpreter for my mum. Even if my grammar might be problematic, the foreigner could understand me. Therefore, I was not nervous anymore and became more confident.”*

At the time of the interview, however, Bei expressed continued concerns about understanding different accents: “*I feel that it may be difficult to understand American native teachers, let alone non-native English speaker teachers. I am worried.”*

Deng, also expressed continued difficulties when engaging in the use of English:

*I did not prepare for oral English, since Chinese often get low scores on oral*… *But if you let me give a talk, especially without preparation I often pause. After that, I realized that I was using wrong words and grammar. That was worse than what I thought*.

Shi described his current state of speaking English as *“scratching my head,”* indicating his continued anxiety.

### Group 3

#### Profile

Of the four members of this group, Miao and Qi, had graduated from a high school in North America and had chosen to continue their studies abroad, attending college. They were juniors, aged 23, at the time of data collection. Lili had spent 10 years abroad and Ning 1 year. This range of duration abroad was typical of this group. Miao’s English proficiency had allowed her to avoid remedial classes in college, but she mostly spoke Chinese in her daily life. In contrast, Qi had lived with a home-stay family while in high school, trying to avoid use of the Chinese language, and had made friends with many English native speakers. Lili, after receiving her bachelor’s degree had stayed in North America to work. She was in her mid 30s and was planning to attend university once again as a graduate student. She was interested in American culture and continued to speak English most of the day. Ning was 25 years old and had received a high enough test score in English on the college entrance exam to skip remedial classes at her university, and had started to learn L2 German. All participants were female.

#### Goal-Directed Activity

Participants had struggled when they first came to study abroad and had tried to improve their English as much as they could. For example, Ning focused on her pronunciation, imitating English native speakers in private rehearsal. At first, Lili, although finding it difficult to communicate in the L2, had persisted in her efforts:

*I forced myself to never give up, try to communicate with English native speakers, watch more TV shows, and so on. I was familiar with the language and culture as well. I tried to search all the information and watched videos on YouTube. That was not quite useful because I found the best way to learn English was speaking out by yourself.*

The extent to which participants were involved in active participation and communication with native speakers varied from person to person. Qi found it easy to adapt to the local community and made many friends. This circumstance was partly due to the low number of Chinese students in her high school, where she stood out as “special,” her classmates curious about her. She believed that this interest was part of the reason why her English improved quickly over her first 3 or 4 months in country. However, once she entered university, she was confronted with stereotypical views of Chinese students, which she found hindered her ability to integrate, “*I do not have much communication with native speakers, only class discussions.*” This concern was also mentioned by Ning, “*I think the local students may not be interested in Chinese students. Additionally, by default, they may think Chinese students like to huddle together.*”

In contrast to Qi and Ning, Miao had chosen a private high school and as based on the large number of Chinese students enrolled, and when at university tried to avoid social interactions in English, concentrating instead on the language for academic purposes, speaking English only during class “*At the beginning, I did not talk to foreigners at all. Now, I talk to them sometimes. If the teachers do not ask me, I will not talk to them, definitely.*” She thought language learning occurred “naturally” and emphasized the importance of living where the language is spoken, despite her own desire not to speak the language to any great extent.

Lili had been communicating in English in her daily life and work and was quite fluent. She held high standards for her use of English because her major was advertising, which is heavily dependent on the use of language. Despite her advanced level of English proficiency, she indicated that it was an on-going struggle to effectively use the language: *“Many of my coworkers are able to talk eloquently. Thus, sometimes when I am with these kinds of people, I feel that they are native Americans and my English is still worse.*”

Two of the other participants besides Lili, Miao and Ning, also believed that native-like proficiency in English would be very difficult to achieve, but that focusing on the language for purposes of graduating and finding suitable employment back in China was within their grasp. Ning was undertaking a 2-year stay in the United States and believed, like Miao, that in order to increase her English proficiency she would need to engage in the use of the language socially, although, in in fact, like Miao, she did not pursue such opportunities. Ning studied Engineering and found that: “*I can understand the content with PPT after class*” which allowed her to progress in the subject despite listening comprehension problems.

Lili additionally reflected on how language learning while in China had been geared to the specifics of the tests administered, affecting goal-directed activity in a misleading way:

*If you stay in China, all you do is for the exam. So, I was an English representative in high school. After I had taken SAT, TOEFL, my English should be improved. Then when I took English tests in China, my score was even worse than before. Thus, I don’t think it is natural enough in the exam-oriented environment. That is different.* (Lili)

#### Affective Reactions

Saving face was a strong motivator for participants in the group. Qi had been especially embarrassed in her home-stay circumstances, which made it painfully apparent to her that initially she had no confidence in her L2 abilities – gains in proficiency were in large part motivated by saving face:

*I was in a home-stay family, the only outsider for the whole family. Everything they were talking about was very oral or authentic. I did not understand what they were talking. Sometimes, it was embarrassing that the whole family was very warm, you know. They really wanted to know more about Chinese culture and talked to you. But I was too nervous to figure what they were asking. I felt so bad about myself at that time, but I had no way out.* (Qi)

Second language confidence is an unavoidable issue for learners, and was more present among those participants who had only been studying abroad for a short period of time. Ning mentioned that *“I am not confident enough about my English and it is difficult to communicate with English native speakers for friendship.”*

Participants also mentioned that learning a new language involved reconstructing the thinking processes. Qi made a conscious effort to change her Chinese “logic” of speaking and tried to learn how to speak English in the way that native speakers do so that her thoughts would follow the logic of the language. This proved to be a transformative process:

*Many things I learned here were different from China. The habitual way of speaking and the logical thinking. I needed to overturn many things I learned from textbooks previously. Sometimes I thought I spoke an advanced sentence to represent my good command of English; however, they [English native speakers] did not speak like that, and expressed in a more casual and logical way.*

In addition, long-term exposure to the target language and culture had influenced participants’ ability to express things verbally in Chinese. Lili and Ning both mentioned the difficulty of describing something in Chinese which they had first learned to talk about in English, especially within their fields of study. Lili also mentioned her emotional changes after having stayed in the United States for 10 years, which was about English learning and identity development.

*When I first came here, I liked it and was happy and proud. Look, how capable I am! After a long time, I realized several problems, feeling insecure sometimes.* (Lili)

### Group 4

#### Profile

Of the three members of this group, Qian and Nana, both females, aged 25, had received master’s degrees abroad after 3 years of study. After coming back to China, Qian had chosen to pursue her doctorate at a university in Beijing, and Nana had chosen to work. Jun, the third interviewee, a 25 year old male, had been an exchange doctoral student, studying in the United States for 1 and a half years before returning to the same university in China to complete his degree.

#### Goal-Directed Activity

The participants did not talk in detail about their use of English in their current circumstances. This was particularly the case for Nana, who did not use English frequently. Jun and Qian mentioned wanting to improve their English as both were doctoral students and continued to read and write in English and to publish in the language as well.

These participants were also asked to reflect on their learning experiences while studying abroad. Jun and Qian said that they had actively sought out opportunities to improve their English. Qian had attended free English classes sponsored by a local church and had interacted with her American roommate, although suggesting that it had been difficult to communicate with her: *“I needed to think for a long time before I talked to her and thought about whether I talked in a right way.”* Jun reported reading a driver’s guide book thoroughly and using a mobile APP to help him remember new vocabulary over a period of more than 200 days. Also, when he withdrew money at a bank, he would go to the counter to talk to a teller instead of using an ATM, saying that: *“I came here in order to speak English”* (said to a bank employee)*.“That lobby man thought this was funny.”* Nana said that although she had had many opportunities to engage socially in English, for example, through attending English clubs or visiting American families, instead she “*chose to focus on English related to the academic work*,” and spent her social time with other Chinese students who had the same major.

Participants also focused on how they had become aware that their English was not as proficient as they would have liked, becoming especially concerned about speaking in public without prior preparation. For instance, Qian mentioned that *“especially after I know their culture, I think some of the English I said before was not rational.”*

#### Affective Reactions

Parental influence was a major factor that had affected English learning experiences for participants across the four groups. For Nana the decision of why to study English and where to study abroad had been determined by her parents. Although many participants in this study had decided to study abroad with advice from their parents, they were enthusiastic about improving their chances for education and career, however, this was not the case for Nana, who felt she had followed her parent’s desires for her, not her own: “*My parents wanted me to study in the U.S. Probably, their generation think that you should go to the U.S. if you study abroad. Nowadays, my father asks me to begin to take the TOEFL to get higher scores, which may be helpful for my work in that company. I don’t know. Whatever, I will take the test if he asks.”*

Qian expressed positive attitudes toward learning English. She had had an interest in learning English in primary school and had organized English study groups to help her classmates prepare for CET 4 and CET 6 exams in China before studying abroad, although thought of English tests in China as tricks to get certificates. When studying abroad, Qian said that she mainly communicated with her advisor, who made her nervous most of the time, pushing her to continue to improve her English:

*My advisor helped me to correct some non-native English. I had a meeting with him every week. After each meeting, I felt good about myself and had a big relief. But sometimes I did not think I said anything useful and that was not what I meant.* (Qian)

Even after studying abroad, Qian and Jun were still not confident speaking English, Qian said she was particularly unhappy that she was still unable to converse freely with others at adamic conferences.

Avoiding the feeling of rejection was brought out by Jun in talking about his English learning experience abroad. He had not been actively engaged in the local community as his motivation had been primarily instrumental, thinking that the study-abroad experience would provide him with better options in the job market after returning, and did not feel any obligation to mix with native speakers:

*I don’t care much whether Americans accept me. I did not try to joint them actively. They are like a bottle of oil, but I am like water. If I want to be mixed, I would feel rejected. But I did not plan to join them, so I don’t feel the rejection*.

## Discussion

The English language learning experience for the Chinese participants in the study in relation to goal-directed activity was found to be connected to the Vygotskian concept of *perezhivanie* as a key aspect of motivation and how interviewees emotionally experienced L2 related events, finding both similarities and differences across members and groups. This perspective is reflective of previous SCT activity studies that tracked changes in orientation longitudinally, a change in *perezhivanie* leading to changes in goal orientation which resulted in a redistribution of activity (Coughlan and Duff, 1994; Lantolf and Swain, 2019; Lantolf and Genung, 2002; Kim, 2009, 2011), if relevant mostly to groups 2, 3, and 4.

While in China, participants in all groups reported similar experiences in learning English up through middle school and into college, a time at which they continued to focus on exercises and drills in preparing for exams as part of progressing toward a desired future through attaining academic goals. A good command of English is viewed in China as a springboard, increasing exam scores and chances to attend leading schools, ultimately helping to land preferred jobs (Magid, 2009; Qin and Dai, 2013; You and Dörnyei, 2014; Yu et al., 2018). Parental expectations play a vital role in meeting such hopes and desires, and as found or one participant in group 4, such expectations can lead to the unwanted course of studying English and then to studying abroad.

Also in line with previous studies (Li, 2014), participants in the EFL group not intending to study abroad (group 1) were not highly motivated to learn the language outside of passing exams and for career preparation as extrinsic goals. Only one participant, Yong, mentioned emergent goals and goal-directed activity as intrinsically motivated. Beyond speaking English for travel purposes, participants suggested wanting to use the language for “showing off” and “translating books.” These are ludic responses, reflecting the lack of relevance that this line of motivational inquiry held for them. English played only a marginal role in their planned futures, remembering however, that Yong as the exception, still had time to decide whether to study abroad or not. Also, differences in regional contexts demonstrated a deficiency of opportunity to study the language as site related, which could have impacted motivation as well.

Participants in group 2, although technically EFL learners, proved more like the ESL groups, the goal of living and studying abroad having already emerged through positive experiences interacting with members of an L2 culture, and so on. They all suggested the importance of environmental exposure and planned to immerse themselves in the target language and culture as part of pursuing academic and professional goals once abroad. Given the enthusiasm displayed by the interviewees toward learning the language and the many goal-directed activities they mentioned, this group clearly had higher levels of positive motivation than group 1. An international posture or desire for involvement in an international community setting has been shown previously as positively affecting English learning experiences (Yashima, 2002, 2009; Kormos et al., 2011; Munezane, 2015; Aubrey and Philpott, 2019).

Participants in group 3, those undergoing a study-abroad experience, expressed motivation as stemming from the need to save face and interact with others in their local contexts, which for those who wanted to improve led to gains in L2 proficiency. Moreover, L2 immersion for Qi and Lili was reported to have affected thinking-for-speaking in the L1 (Qi and Lili). In contrast, the other two participants (Miao and Ning) chose not to interact socially in the L2, instead concentrating on the language primarily for academic purposes, which also determined goal-directed activity. For all participants in this group the L2 environment should be considered to have played an important role in relation to both motivation and *perezhivanie* and driving emergent activity.

Participants in group 4, after returning from their study-abroad experience, said little about the pursuit of specific goal-directed activity either in relation to maintaining current levels of L2 proficiency or increasing levels of proficiency. Instead, these participants were oriented toward their past study-abroad experiences, which mirrored much of what group 3 reported. It may have been that future self was now limited to what they thought they could actually achieve in the language together with having realized that “perfect” English was not needed to be an effective communicator. Further L2 study for them was relegated to possible future employment possibilities, when it might be needed again, their position in life having been settled for the moment, L2 goals no longer having the same impact as they had once had.

## Conclusion

The current study examined the dynamic interaction of motivation and emotional experience for Chinese learners of English in four different contexts of L2 exposure as affecting the language learning process. From the SCT activity perspective taken in the study, motivation was found to shape experience and experience was found to shape motivation, the two intertwining in accordance with expectations, both personal and cultural, what they experienced and how this affected the forms of activity they pursued. Findings demonstrated both within and across group similarities and differences and recognized both the dominant role of culture and the importance of considering each person as an individual.

By choosing to examine L2 learners in different contexts of exposure the study was able to gain insights into how learning environments affect language learning. Future research would benefit from following participants over time in any one context to further understanding of the interrelation/interaction of motivation, *perezhivanie* and goal-directed activity as a critical aspect of the dynamic nature of the L2 learning process as informing both applied linguistics and education as a whole.

## Data Availability Statement

The original contributions presented in this study are included in the article. Further inquiries can be directed to the corresponding authors.

## Ethics Statement

The studies involving human participants were reviewed and approved by the University of Nevada, Las Vegas Office of Research Integrity – Human Subjects. The patients/participants provided their written informed consent to participate in this study.

## Author Contributions

LT designed the project, collected and analyzed the data, and worked on the draft writing and revision. SM helped to design the project, analyzed the data, and revised the drafts. Both authors contributed to the article and approved the submitted version.

## Conflict of Interest

The authors declare that the research was conducted in the absence of any commercial or financial relationships that could be construed as a potential conflict of interest.

## Publisher’s Note

All claims expressed in this article are solely those of the authors and do not necessarily represent those of their affiliated organizations, or those of the publisher, the editors and the reviewers. Any product that may be evaluated in this article, or claim that may be made by its manufacturer, is not guaranteed or endorsed by the publisher.

## References

[B1] Aragão R. (2011). Beliefs and emotions in foreign language learning. *System* 39, 302–313.

[B2] Arievitch I. M. (2017). *Beyond the Brain: An Agentive Activity Perspective on Mind, Development, and Learning.* Berlin: Springer. 10.1007/978-94-6351-104-9

[B3] Aubrey S. Philpott A. (2019). Inter-cultural and Intra-cultural contact and the L2 motivational self system: an EFL classroom intervention study. *RELC J.* 52 440–457. 10.1177/0033688219865409

[B4] Bigelow M. (2019). (Re) considering the role of emotion in language teaching and learning. *Mod. Lang. J.* 103, 515–516. 10.1111/modl.12569

[B5] Benesch S. (2019). Exploring emotions and power in L2 research: sociopolitical approaches. *Mod. Lang. J.* 103, 530–533. 10.1111/modl.12575

[B6] Bielak J. Mystkowska-Wiertelak A. (2020). Language teachers’ interpersonal learner-directed emotion-regulation strategies. *Lang. Teach. Res.* 1362168820912352. 10.1177/1362168820912352

[B7] Blunden A. (2016). Translating perezhivanie into English. *Mind Cult. Act.* 23 274–283. 10.1080/10749039.2016.1186193

[B8] Chang H-c (2018). Exploring non-English majors’ EFL learning motivational dynamics—case studies from the perspective of DST. *Technol. Enhanc. Foreign Lang.* 5 35–41.

[B9] Chen A. Wu B. (2011). “The regional division of the higher education sector in China: a spatial analysis,” in *Higher Education Reform in China: Beyond the Expansion*, eds Morgan W. J. Wu B. (Oxfordshire: Routledge), 13–29.

[B10] Chen J. F. Warden C. A. Chang H. T. (2005). Motivators that do not motivate: the case of Chinese EFL learners and the influence of culture on motivation. *Tesol Q.* 39 609–633. 10.2307/3588524

[B11] Coughlan P. Duff P. A. (1994). “Same task, different activities: analzysis of a SLA task from an activity theory perspective,” in *Vygotskian Approaches to Second language research*, eds Lantolf J. Appel G. (Norwood, NJ: Ablex), 173–193.

[B12] Creswell J. W. (2007). *Educational Research: Planning, Conducting, and Evaluating Quantitative and Qualitative Research*, 3rd Edn. Los Angeles, CA: Sage.

[B13] Cross R. (2012). Creative in finding creativity in the curriculum: The CLIL second language classroom. *Aust. Educ. Res.* 39 431–445. 10.1007/s13384-012-0074-8

[B14] De Bot K. (2008). Introduction: second language development as a dynamic process. *Modern Lang. J.* 92 166–178. 10.1111/j.1540-4781.2008.00712.x

[B15] De Bot K. Lowie W. Verspoor M. (2007). A dynamic systems theory approach to second language acquisition. *Biling. Lang. Cogn.* 10 7–21. 10.1017/S1366728906002732

[B16] Dewaele J.-M. (2015). On emotions in foreign language learning and use. *Lang. Teach.* 39, 13–15.

[B17] Dewaele J. M. (2019). When elephants fly: the lift-off of emotion research in applied linguistics. *Mod. Lang. J.* 103, 533–536.

[B18] Dörnyei Z. (2009). “The L2 motivational self system,” in *Motivation, Language Identity and the L2 Self*, eds Do r̈nyei Z. Ushioda E. (Bristol: Multilingual Matters), 9–42. 10.21832/9781847691293-003

[B19] Dörnyei Z. MacIntyre P. D. Henry A. (2015). “Introduction: applying complex dynamic systems principles to empirical research on L2 motivation,” in *Motivational Dynamics in Language Learning*, eds Dornyei Z. MacIntyre P. D. Henry A. (Bristol: Multilingual Matters), 1–7. 10.21832/9781783092574-003

[B20] Dörnyei Z. Ryan S. (2015). *The Psychology of the Language Learner Revisited.* Oxfordshire: Routledge. 10.4324/9781315779553

[B21] Ferholt B. Nilsson M. (2016). Perezhivaniya as a means of creating the aesthetic form of consciousness. *Mind Cult. Act.* 23 294–304. 10.1080/10749039.2016.1186195

[B22] Gao Y. Zhao Y. Cheng Y. Zhou Y. (2004). Motivation types of Chinese university undergraduates. *Asian J. English Lang. Teach.* 14 45–64.

[B23] Garratt D. A. (2012). *Students’ Perceptions of the Use of Peer-to-Peer ESL Text Chat: An Introductory Study.* Doctoral dissertation. Albuquerque, NM: The University of New Mexico.

[B24] Heying A. Kennedy F. (2016). Chinese and Irish students: an investigation of their intercultural competence and second language learning motivation in the process of integration. *Call Irish J. Cult. Arts Lit. Lang.* 1:7.

[B25] Imai Y. (2010). Emotions in SLA: new insights from collaborative learning for an EFL classroom. *Mod. Lang. J.* 94, 278–292.

[B26] Kim T. Y. (2009). “The sociocultural interface between ideal self and ought-to self: a case study of two Korean students’ ESL motivation,” in *Motivation, Language Identity and the L2 Self*, eds Dornyei Z. Ushioda E. (Bristol: Multilingual Matters), 274–294. 10.21832/9781847691293-015

[B27] Kim T. Y. (2011). Sociocultural dynamics of ESL learning (de) motivation: an activity theory analysis of two adult Korean immigrants. *Can. Modern Lang. Rev.* 67 91–122. 10.3138/cmlr.67.1.091

[B28] Kong K. (2019). Embracing an integrative approach toward emotion in language teaching and learning. *Mod. Lang. J.* 103, 539–544.

[B29] Kormos J. Kiddle T. Csizér K. (2011). Systems of goals, attitudes, and self-related beliefs in second-language-learning motivation. *Appl. Linguist.* 32 495–516. 10.1093/applin/amr019

[B30] Kozulin A. (2016). The mystery of perezhivanie. *Mind Cult. Act.* 23 356–357. 10.1080/10749039.2016.1230134

[B31] Lantolf J. P. Genung P. (2002). “I’d rather switch than fight”: an activity-theoretic study of power, success, and failure in a foreign language classroom,” in *Language Acquisition and Language Socialization: Ecological Perspectives*, ed. Kramsch C. (London: Continuum), 175–196.

[B32] Lantolf J. P. Swain M. (2019). “4. Perezhivanie: the cognitive–emotional dialectic within the social situation of development,” in *Contemporary Language Motivation Theory*, eds MacIntyre P. D. Al-Hoorie A. H. (Bristol: Multilingual Matters), 80–106. 10.21832/9781788925211-009

[B33] Li C. Ruan Z. (2015). Changes in beliefs about language learning among Chinese EAP learners in an EMI context in Mainland China: a socio-cultural perspective. *System* 55 43–52. 10.1016/j.system.2015.08.010

[B34] Li Q. (2011). *The Motivation of Chinese Learners of English in a Foreign and Second Language Context.* Auckland: ResearchSpace@.

[B35] Li Q. (2014). Differences in the motivation of Chinese learners of English in a foreign and second language context. *System* 42 451–461. 10.1016/j.system.2014.01.011

[B36] Lichtman M. (2012). *Qualitative Research in Education: A User’s Guide: A user’s Guide.* Thousand Oaks, CA: Sage.

[B37] Liu M. (2007). ‘Chinese students’ motivation to learn English at the tertiary level. *Asian EFL J.* 9 126–146. 26233678

[B38] Magid M. (2009). The L2 motivational self system from a Chinese perspective: a mixed methods study. *J. Appl. Linguist.* 6 69–90. 10.1558/japl.v6i1.69

[B39] Mahn H. John-Steiner V. (2002). “The gift of confidence: a vygotskian view of emotions,” in *Learning for Life in the 21st Century*, eds Wells G. Claxton G. (Oxford: Blackwell), 46–58.

[B40] Munezane Y. (2015). Enhancing willingness to communicate: relative effects of visualization and goal setting. *Modern Lang. J.* 99 175–191. 10.1111/modl.12193

[B41] Pavelescu L. M. (2019). Motivation and emotion in the EFL learning experience of Romanian adolescent students: two contrasting cases. *Stud. Sec. Lang. Learn. Teach.* 9 55–82. 10.14746/ssllt.2019.9.1.4

[B42] Peltier I. N. McCafferty S. G. (2010). Gesture and identity in the teaching and learning of Italian. *Mind Cult. Act.* 17 331–349. 10.1080/10749030903362699

[B43] Poehner M. E. Swain M. (2016). L2 development as cognitive-emotive process. *Lang. Sociocult. Theory* 3, 219–241.

[B44] Poole A. Huang J. (2018). Resituating funds of identity within contemporary interpretations of perezhivanie. *Mind Cult. Act.* 25, 125–137.

[B45] Prior M. T. (2019). Elephants in the room: an “Affective Turn,” or just feeling our way? *Mod. Lang. J.* 103, 516–527.

[B46] Qin L. Dai W. (2013). A study on college English learning motivation self system model framed by the activity theory. *Foreign Lang. World* 6 23–31.

[B47] Roth W. M. Lee Y. J. (2007). Vygotsky’s neglected legacy”: cultural-historical activity theory. *Rev. Educ. Res.* 77 186–232. 10.3102/0034654306298273

[B48] Taguchi T. Magid M. Papi M. (2009). “The L2 motivational self system among Japanese, Chinese and Iranian learners of English: a comparative study,” in *Motivation, Language Identity and the L2 self*, eds Dornyei Z. Ushioda E. (Bristol: Multilingual Matters), 66–97. 10.21832/9781847691293-005

[B49] Tian L. McCafferty S. G. (2021). Chinese international students’ multicultural identity and second language development: gesture awareness and use. *Lang. Aware.* 30 114–133. 10.1080/09658416.2020.1767118

[B50] Vygotsky L. S. (1978). “Interaction between learning and development,” in *Mind in Society: The Development of Higher Psychological Processes*, eds Cole M. John-Steiner V. Scribner S. Souberman E. (Cambridge, MA: Harvard University Press).

[B51] Vygotsky L. S. (1994). “The problem of the environment. (Orig. 1935),” in *The Vygotsky Reader*, eds Van der Veer R. Valsiner J. (Oxford: Blackwell), 338–354.

[B52] Wang Y. Derakhshan A. Zhang L. J. (2021). Researching and practicing positive psychology in second/foreign language learning and teaching: the past, current status and future directions. *Front. Psychol.* 12:731721. 10.3389/fpsyg.2021.731721 34489835 PMC8417049

[B53] Waninge F. Dörnyei Z. De Bot K. (2014). Motivational dynamics in language learning: change, stability, and context. *Modern Lang. J.* 98 704–723. 10.1111/modl.12118

[B54] Warden C. A. Lin H. J. (2000). Existence of integrative motivation in an Asian EFL setting. *Foreign Lang. Ann.* 33 535–545. 10.1111/j.1944-9720.2000.tb01997.x

[B55] Wu Y. A. (2001). ‘English language teaching in China: trends and challenges,’. *TESOL Q.* 35 191–194. 10.2307/3587867

[B56] Yashima T. (2002). Willingness to communicate in a second language: the Japanese EFL context. *Modern Lang. J.* 86 54–66. 10.1111/1540-4781.00136

[B57] Yashima T. (2009). “International posture and the ideal L2 self in the Japanese EFL context,” in *Motivation, Language Identity and the L2 Self*, Vol. 86 eds Dörnyei Z. Ushioda E. (Clevedon, UK: Multilingual Matters), 144–163. 10.21832/9781847691293-008

[B58] Yasnitsky A. van der Veer R. (eds) (2016). *Revisionist Revolution in Vygotsky Studies.* New York, NY: Routledge/Taylor & Francis Group. 10.4324/9781315714240

[B59] You C. J. Dörnyei Z. (2014). Language learning motivation in China: results of a large-scale stratified survey. *Appl. Linguist.* 37 495–519. 10.1093/applin/amu046

[B60] You C. J. Dörnyei Z. Csizér K. (2016). Motivation, vision, and gender: a questionnaire of learners of English in China. *Lang. Learn.* 66 94–123. 10.1111/lang.12140

[B61] Yu J. Brown G. T. Stephens J. M. (2018). Retrospective case studies of successful Chinese learners of English: continuity and change in self-identities over time and across contexts. *System* 72 124–138. 10.1016/j.system.2017.11.008

[B62] Yuan S. Ji Y. (2017). Case studies of college students’ dynamic characteristics of in-class English learning motivation. *Foreign Lang. World* 48–56.

